# Supplementary material for: Discrepancy of C-Reactive Protein, Procalcitonin and Interleukin-6 at Hospitalization: Infection in Patients with Normal C-Reactive Protein, Procalcitonin and High Interleukin-6 Values
Source: J Clin Med. 2022 Dec 9;11(24):7324. doi: 10.3390/jcm11247324 (PMC9783053; doi:10.3390/jcm11247324)
Supplement: Supplementary file 1 [file jcm-11-07324-s001.zip › jcm-2040114-supplementary.pdf]

Table S1. Baseline characteristics of the discrepancy and follow-up dataset.

Characteristics	Discrepancy Dataset	Follow-Up Dataset
Number of total patients	7149	1827
Re-admission	0	118
ER visit	6725 (94.1)	1855 (95.4)
Total number of simultaneous tests	7149	5261
Bacteremia or fungemia [a]	597 (8.4)	268 (14.7)
30-day all-cause in-hospital mortality	672 (9.4)	285 (15.6)
**Trimmed data**		
CRP		
< 0.3 mg/L	3 (0.04)	24 (0.34)
Procalcitonin		
> 400 ng/mL	3 (0.04)	5 (0.07)
< 0.02 ng/mL	79 (1.11)	55 (0.77)
IL-6		
> 5000 pg/mL	253 (3.54)	181 (2.53)
< 1.5 pg/mL	162 (2.27)	46 (0.64)
**Major symptoms or sings of hospitalization** [b]		
Fever, chilling	2611 (36.5)	594 (30.5)
General weakness, poor oral intake	1330 (18.6)	254 (13.1)
Dyspnea, DOE, SOB	957 (13.4)	387 (19.9)
Abdominal pain, distension, vomiting	659 (9.2)	211 (10.8)
Mental change, loss of consciousness, seizure	539 (7.5)	169 (8.7)
Chest pain, discomfort	486 (6.8)	25 (1.3)
Trauma, musculoskeletal pain, wound problems	452 (6.3)	96 (4.9)
Hematemesis, hematochezia and melena	377 (5.3)	127 (6.5)
Focal neurologic signs [c], dysarthria	305 (4.3)	101 (5.2)
Syncope, collapse	287 (4.0)	164 (8.4)
Urinary symptom [d]	197 (2.8)	87 (4.5)
Skin rash	160 (2.2)	17 (0.9)
General edema	138 (1.9)	38 (2.0)
Hemoptysis, blood-tinged sputum	84 (1.8)	21 (1.1)

Data were expressed as number (percent) or median (interquartile range). [a] It means-a case in which the microorganisms were identified in the blood cultures conducted within 3 days of admission. [b] Several patients had the multiple reasons. [c] Include weakness of extremities and paralysis or sensory change. [d] Include urinary retention, incontinence, frequency, urgency, dysuria or hematuria. Aberrations: CRP, C-reactive protein; DOE, dyspnea on exertion; ER, emergency room; IL, interleukin; SOB, shortness of breath.

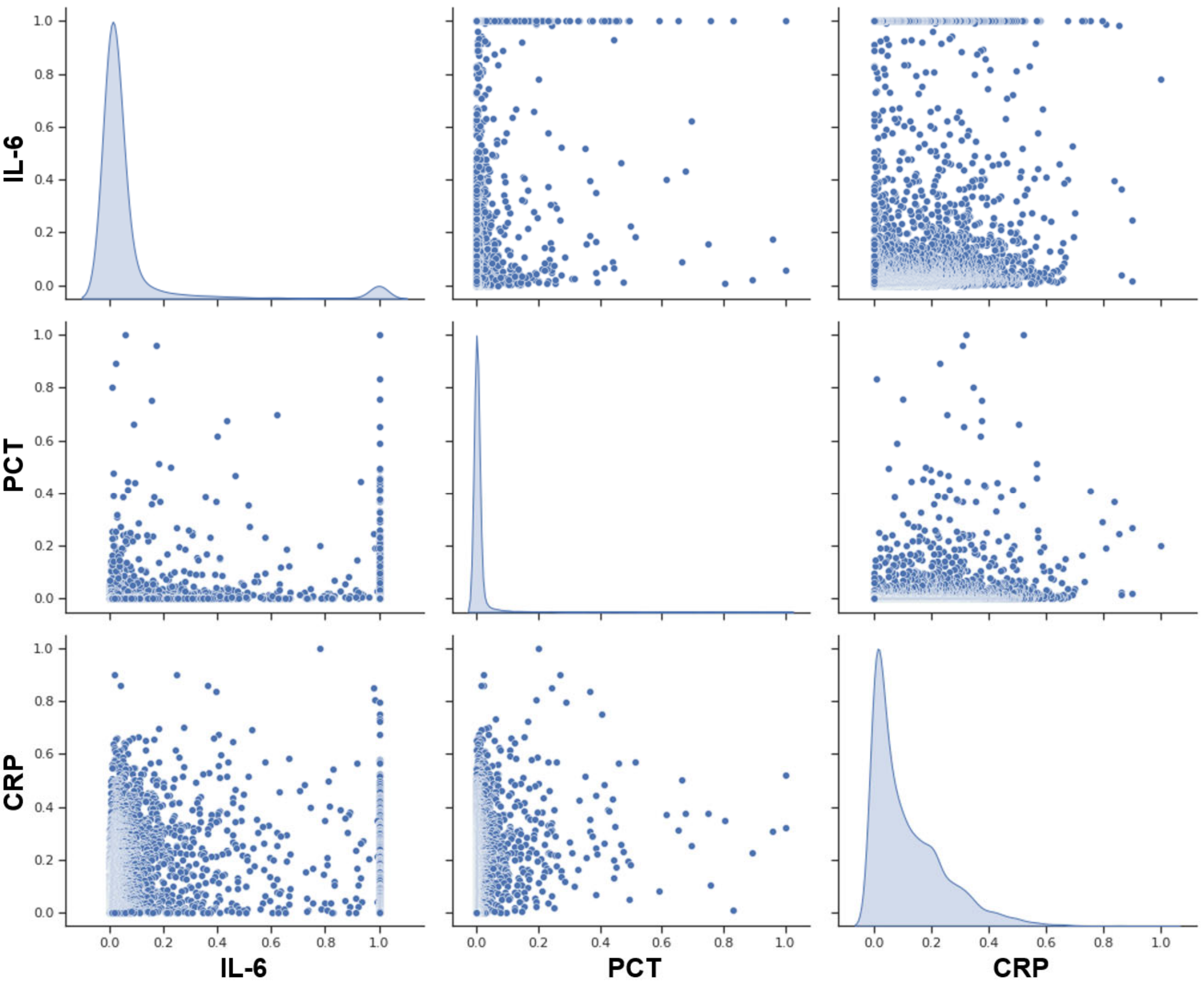

Figure S1. Pair plots of the normalized CRP, PCT, and IL-6 values (discrepancy dataset). The three graphs located on the diagonal of the figure show the distribution of each of CRP, PCT, and IL-6 obtained by the kernel density estimation method.

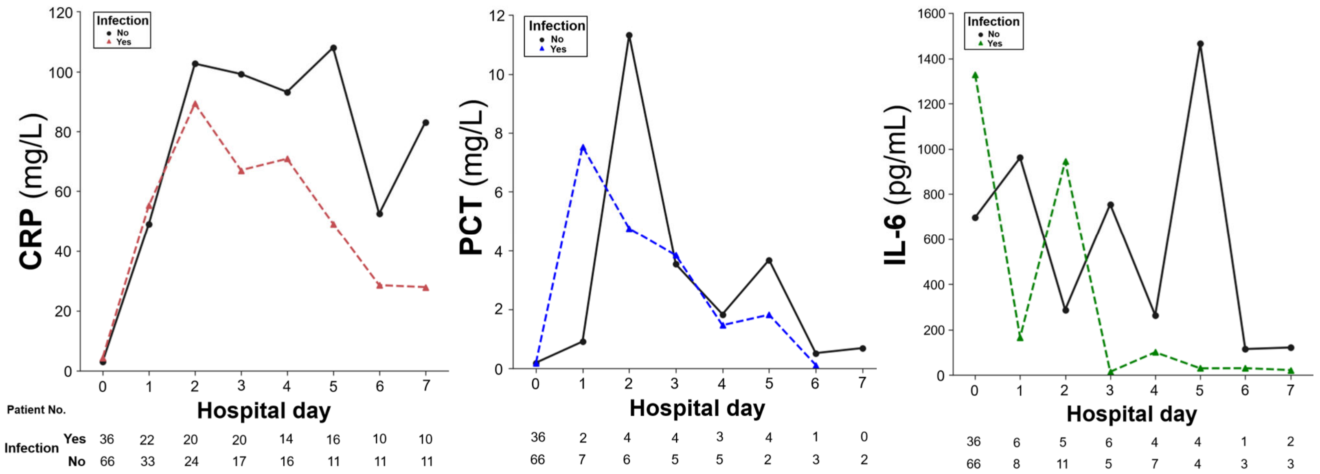

Figure S2. Changes of three inflammatory markers in patients with normal CRP and procalcitonin but high IL-6 levels within 24 hours of admission according to presence of infection. Each point represents the average of the measured values.
